# Risk Factors for and Barriers to Control Type-2 Diabetes among Saudi Population

**DOI:** 10.5539/gjhs.v8n9p10

**Published:** 2015-10-16

**Authors:** Yahya Mari Alneami, Christopher L. Coleman

**Affiliations:** 1 School of Nursing and Health Sciences, La Salle University, Philadelphia, USA; 2 School of Nursing, University of Pennsylvania, Philadelphia, USA

**Keywords:** barrier, risk factor, environment, Saudi Arabia, Type-2 Diabetes

## Abstract

**Background:**

The prevalence of Type-2 Diabetes is dramatically increasing in urban areas within Saudi Arabia. Hence, Type-2 Diabetes has now become the most common public health problem. Understanding the major risk factors for and barriers to control Type-2 Diabetes may lead to strategies to prevent, control, and reduce in the burden of disease cases.

**Objective:**

To describe risk factors for and barriers to control Type- 2 Diabetes in Saudi Arabia.

**Methods:**

The literature search was conducted on risk factors for and barriers to control Type- 2 Diabetes in Saudi Arabia using the databases PubMed, MEDLINE, and Google Scholar (2007-2015). The literature search yielded 80 articles, of which 70 articles were included in this review after excluding non-relevant articles.

**Results:**

The literature review revealed that obesity, physical inactivity, unhealthy diet, smoking, and aging are the major risk factors for Type-2 Diabetes in Saudi Arabia. Further, the review allocated a complex set of barriers including, lack of education, social support, and healthy environment. These barriers may hinder Saudis with Type-2 Diabetes from controlling their disease.

**Conclusion:**

The prevalence of Type-2 Diabetes is high among the Saudi population and represents a major public health problem. Effective research programs are needed to address the modifiable risk factors for and barriers to control Type-2 Diabetes among Saudi population.

## 1. Introduction

Type-2 Diabetes (T2D) is a disease that results when the body either doesn’t make enough insulin or cannot use its own insulin properly (The American Diabetes Association [ADA], 2015). According to Centers of Disease Control and Prevention [CDC], 2014) diabetes is the leading cause of severe health complications including heart disease, blindness, kidney failure, and nontraumatic lower limb amputations. Further, there are 347 million people living with diabetes globally, and T2D accounts for almost 90% of all cases of diabetes in the world (A. B. Olokoba, Obateru, & L. B. Olokoba, 2012). Additionally, the prevalence of T2D will dramatically increase worldwide.

It is estimated that the number of people with diabetes will increase to 439 million by 2030 and to 592 million by 2035 (Guariguata et al., 2014; Shaw, Sicree, & Zimmet, 2010). Further, Assaad-Khalil et al. (2013) reported that the growing burden of diabetes, particularly in developing countries is a public health concern. According to the International Diabetes Federation, over 70% of people with T2D are from developing countries (Rawal et al., 2012). Additionally, between 2010 and 2030, it was estimated that there would be a 69% increase in the number of people with diabetes in developing countries compared to only a 20% increase in developed countries (Shaw, Sicree, & Zimmet, 2010). Unfortunately, Saudi Arabia is one of the developing countries that has a very high prevalence of T2D (Assaad-Khalil et al., 2013).

International Diabetes Federation reported that Saudi Arabia is one of the top 10 countries with the highest prevalence of diabetes among adults aged 20 to 79 years (Boutayeb, Boutayeb, Lamlili, & Boutayeb, 2013). Further, data indicated the prevalence of T2D among Saudi population sharply increased from 7% in 1989 to 32% in 2009 (Alharbi et al., 2014). These statistics highlight the importance of preventing and controlling T2D in Saudi Arabia. The purpose of this paper is to describe risk factors for and barriers to control T2D in Saudi Arabia.

## 2. Search Strategy Methods

The literature search was conducted on risk factors for and barriers to control Type-2 Diabetes in Saudi Arabia using the databases PubMed, MEDLINE, and Google Scholar (2007-2015). The literature search yielded 80 articles, of which 70 articles were included in this review after excluding non-relevant articles.

## 3. Findings of the Literature Review

### 3.1 Diabetes Risk Factors

T2D is one of the quickest growing public health issues worldwide (Olokoba et al., 2012; Qi, Hu, & Hu, 2008; Rawal et al., 2012). In order to control T2D, it is necessary to determine associated risk factors (Murad et al., 2014). Detecting major risk factors for T2D can lead to approaches that prevent, control, and reduce in the burden of disease cases (Murad et al., 2014). Studies reported that obesity, physical inactivity, unhealthy diet, and smoking are risk factors for developing T2D in Saudi Arabia (AlQuaiz, & Tayel, 2009; Lindström et al., 2013; Midhet, Al-Mohaimeed, & Sharaf, 2010b). Further, studies underscored that aging is also a risk factor for T2D in Saudi Arabia (Albakr, Mohammad, Mohammed, & Khamis, 2013; Al-Nozha et al., 2007).

#### 3.1.1 Obesity

Obesity is a disorder characterized by excessive accumulation of adipose tissue in human body (Memish et al., 2014). Several studies showed that obesity is one of the common modifiable risk factors associated with increase risk of T2D (Memish et al., 2014; Murad et al., 2014). Additionally, obesity is the most important factor in causing insulin resistance (Murad et al., 2014). Further, it was reported that 80 to 90% of people with T2D are obese (Aljohani, 2014; Sidawi, Alhariri, & Albaker, 2014). Unfortunately, obesity rate in Saudi Arabia is high.

For example, Al-Quwaidhi, Pearce, Critchley, Sobngwi, and O’Flaherty (2014) conducted a secondary analysis of published data to estimate the trends and projections in the prevalence of adult obesity in Saudi Arabia over the period 1992-2022. In this study, Al-Quwaidhi et al. (2014) reported that the prevalence of obesity among adults in Saudi Arabia increased from 22% in 1993 to 36% in 2005, and it is expected to reach to 41% and to 78% by 2022 in men and women respectively. Additionally, Murad et al. (2014) revealed that the increasing incidence of T2D in the Saudi population is linked to obesity. Similarly to obesity, physical inactivity in Saudi Arabia clearly influences the development of T2D.

#### 3.1.2 Physical Inactivity

The association between physical inactivity as a risk factor for T2D was assessed in many studies (Alqurashi et al., 2011; Al-Quwaidhi, Critchley, Flaherty, & Pearce, 2013; Amin et al., 2014; Hu, 2011; Qi et al., 2008; Midhet et al., 2010a; Temelkova-Kurktschiev & Stefanov, 2012). Unfortunately, lack of physical activity is common among the Saudi population (Al-Nozha et al., 2007; Amin et al., 2014; Amin, Al Khoudair, Al Harbi, & Al Ali, 2012; Midhet et al., 2010a; Sidawi et al., 2014). For example, AlQuaiz and Tayel (2009) conducted a cross-sectional study on 450 Saudi participants at King Khalid University Hospital (KKUH) in Riyadh city and reported the prevalence of physical inactivity among the participants was 82%. In this study, AlQuaiz & Tayel (2009) also underscored that females were more physically inactive 88% compared to males 72%.

Additionally, Al-Nozha et al. (2007) conducted a community-based national epidemiological health survey; enrolled 17395 Saudi males and females aged 30-70 years and reported that 93% of Saudi male participants and 98% of Saudi female participants were physically inactive. In addition, unhealthy diet is a major risk factor for T2D in Saudi Arabia.

#### 3.1.3 Unhealthy Diet

Several studies disclosed that diet is involved in the development of T2D (Aida, Heather, Maria, & Peter, 2013; Alqurashi, Aljabri, & Bokhari, 2011; Midhet et al., 2010b; Temelkova-Kurktschiev & Stefanov, 2012). Adapting Western dietary patterns including unhealthy dietary habits comprising of high fat and sugar is a significant factor and responsible for the progress of T2D (Al-Khudairy, Stranges, Kumar, Al-Daghri, & Rees, 2013; Midhet et al., 2010a; Temelkova-Kurktschiev & Stefanov, 2012).

For instance, a cohort study was conducted in the United States (US), revealed that the risk of being diagnosed with T2D was significantly higher among those who adapted Western dietary patterns, characterized by higher consumption of meat, French fries, and dessert compared to those having prudent dietary patterns including fresh vegetables and fruits and whole grain (Midhet et al., 2010b). Further, in Saudi Arabia, several data revealed that consumption of unhealthy diet significantly increased the risk of T2D among Saudi population (Al-Khudairy et al., 2013; Midhet et al., 2010b; Temelkova-Kurktschiev & Stefanov, 2012). Unfortunately, unhealthy dietary habits are quite common among the Saudi population.

Certain dietary patterns are homegrown in Saudi Arabia including the consumption of dates, desserts, and meat over rice dishes which contains a high content of fat and carbohydrates (Al-Khudairy et al., 2014; Midhet et al., 2010b). Additionally, Sidawi et al. (2014) reported a quarter of Saudis did not consume the recommended healthy food such as fruits and vegetables, and also quarter of them consumed a lot of unhealthy foods and drinks including French fries, donuts, and energy drinks. In addition to unhealthy diet, smoking is also a risk factor for T2D.

#### 3.1.4 Smoking

The association of smoking with T2D was examined in multiple studies (Amin et al., 2014; Eze et al., 2014; Hu, 2011; Luo et al., 2013; Jee, Foong, Hur, & Samet, 2010; Murad et al., 2014; Saeed, 2012; Zhang, Curhan, Hu, Rimm, & Forman, 2011). Smoking is associated with glucose intolerance, impaired fasting glucose, and worsened diabetes control (Saeed, 2012). In addition, the CDC (2014) reported that smoking caused T2D and indicated that smokers are 30–40% more likely to develop T2D than nonsmokers.

Jee et al. (2010) conducted a 14-year prospective cohort study on 1,236,443 Korean men and women, aged 30–95 and concluded that smoking was significantly associated with increased risk for T2D. Jee et al. (2010) also reported that male smoker participants who smoked ≥20 cigarettes/day had increased their risk for T2D compared with never smoker participants. Unfortunately, smoking is more prevalent in Saudi Arabia.

Over 15 billion cigarettes are puffed away every year in Saudi Arabia, making the country the fourth largest importer of manufactured tobacco in the world (Bassiony, 2009). Further, the prevalence of smoking is high in Saudi Arabia at different age groups. For instance, the median prevalence rate of current smoking in Saudi Arabia is 17% among high school students, 14% among university students, 23% among adults, and 25% among elderly people (Bassiony, 2009). In addition, the association between smoking and T2D was studied in Saudi Arabia.

For example, Saeed (2012) conducted a community-based cross-sectional study on 4654 Saudi subjects. In this study the smoking habits were self reported by subjects themselves and were not biochemically verified. Saeed (2012) underscored that there was a significant association between smoking habits and diabetes and also highlighted that diabetic participants were significantly more daily smokers than non-diabetics. Additionally, aging represents as non-modifiable risk factor for developing T2D in Saudi Arabia.

#### 3.1.5 Aging

The prevalence of T2D is high in elderly, a population that is increasing (Weinger, Beverly, & Smaldone, 2014). Additionally, data indicate that diabetes prevalence increases with age (Lee, & Park, 2014; Ng, Zaghloul, Ali, Harrison, & Popkin, 2011). It is reported that 38% of people 65 years and older have diabetes (Lee, & Park, 2014). In Saudi Arabia, aging is significantly associated with the presence of diabetes (Albakr et al., 2013). A cross-sectional study on 691 Saudi employees aimed to assess the prevalence of Non Communicable Diseases (NCDs) risk factors including T2D, indicated that the prevalence of NCDs was higher among those older than 50 years compared to those less than 50 years old (Amin et al., 2014).

Type-2 Diabetes is a serious disease that prevalent in a large part of Saudi Arabia. The Saudi population is at greater risk for developing T2D because of the above risk factors. However, there are a number of obstacles still exist. In order to eliminate the prevalence of T2D, it is necessary to identify barriers and address them effectively.

### 3.2 Barriers to Control Diabetes

Describing barriers to diabetes control is a vital step in attaining best possible health outcomes (Abdelmarouf, Alzohairy, & Hasan, 2011). Healthy People 2020 indicate that factors such as education, social support, and availability of community-based resources in support of community living and opportunities for recreational activities are needed in order to promote a good health (U.S. Department of Health and Human Services, 2014). These factors are known to influence the management of diabetes as well (Al-Khudairy et al., 2014; Schiøtz, Bøgelund, Almdal, Jensen, & Willaing, 2012). In Saudi Arabia, lack of education, social support, and healthy environments may hinder individuals with T2D from controlling their disease.

#### 3.2.1 Education and Diabetes

Knowledge and awareness about diabetes, its risk factors, complications, and management are important aspects for better control and better quality of life (Abdelmarouf et al., 2011). On the other hand, lack of knowledge about risk factors for diabetes hampers preventive efforts and diabetes management such as the adoption of positive lifestyle changes (Aljoudi, & Taha, 2009). Further, education is important to adopting a healthy lifestyle in Saudi Arabia (Al-Khudairy et al., 2014; Midhet et al., 2010a, 2010b).

For example, Saudis with a high school education or lower are more likely to have poor dietary habits and lack physical activity compared to a university educational level (Midhet et al., 2010b). Moreover, lack of knowledge about diabetes is one of the strongest determinants of misconceptions among Saudis with T2D. Al-Khudairy et al. (2014) carried out a cross sectional study on108 Saudis of both genders with or without T2D and reported that dietary misconceptions were higher among diabetics (17%) in comparison to non-diabetics (4%). Al-Khudairy et al. (2014) also disclosed that 83% of diabetic participants thought snacking was essential for diabetics, and 66% thought bitter food would normalize their hyperglycemia.

Additionally, diabetes is a curable disease, diabetes is only a genetic disease, one can feel if blood sugar increases, diabetes treatments cannot prevent complications, and there is no need to take medicines when blood sugar is normal are common misconceptions among Saudis with T2D (Abdelmarouf et al., 2011; Alsunni, Albaker, & Badar, 2014). Beside these misconceptions, social support also influences diabetes management in Saudi Arabia.

#### 3.2.2 Social Support and Diabetes

Social support is defined as an exchange of resources between at least two individuals, aimed at increasing the well being of the receiver (Kadirvelu, Sadasivan, & Ng, 2012). Good social support is significantly associated with health promoting behaviors and well being among individuals with T2D (Mayberry, & Osborn, 2012; Miller & DiMatteo, 2013; Schiøtz et al., 2012). Further, family members can have a positive and/or negative impact on the health of individuals with diabetes and impede with or facilitate self-care activities such as buying groceries or refilling prescriptions (Mayberry, & Osborn, 2012). Studies were conducted on the positive association between high social support and diabetes management (Kadirvelu et al., 2012; Miller & DiMatteo, 2013).

For example, Keyvanara, Hosseini, & Emami, (2012) applied a descriptive analytic method to find a relation between social support and diabetes control among 320 patients with T2D and reported that the patients who had extremely high social support (emotional, instrumental, and informational) could successfully control their diabetes compared to those with low social support. In Saudi Arabia data showed that lack of social support is a significant barrier to adapting healthy lifestyles among individuals with T2D (Al-Khudairy et al., 2014).

AlQuaiz and Tayel (AlQuaiz, & Tayel, 2009) disclosed that the large numbers of social gatherings with extended families interfere with adherence to a healthy diet. Saudis usually insist their guests to over consume during social occasions and this may lead Saudi with T2D to struggles to relinquish preferred foods and substitute with healthy (Al-Khudairy et al., 2014). In addition, lack of social support is a barrier to physical activity. AlQuaiz and Tayel (2009) highlighted that lack of social support was reported by 77% of study group to be a barrier for adherence to physical activity in Saudi Arabia. Similarly to lack of social support, lack of healthy environments impacts the management of T2D.

#### 3.2.3 Healthy Environments and Diabetes

Controlling diabetes is more than simply advising people to lose weight, eat healthy food, and exercise regularly. Environment significantly contributes to the lifestyle and habits of its citizens, including chance for exercise, food, rest, and sleep (Pasala, Rao, & Sridhar, 2010; Sidawi, & Deakin, 2013). Unhealthy environment, which is characterized by lacking of public pools, recreation centers, cycling infrastructures, physical fitness facilities, parks, and sidewalks may prevent individuals from controlling T2D (Allanah, Ashley, & Farley, 2010; Pasala et al., 2010; Salois, 2012). Further, physical structures surroundings such as grocery stores and pleasant opportunities for physical activity can shape peoples’ health (Allanah et al., 2010).

Food environment, which includes supermarkets, food stands, convenience stores, and restaurants can differ by neighborhoods and influence health outcomes. For example, Larson, Story, and Nelson, (2009) revealed that American residents with more access to grocery stores and limited access to fast food generally had better diets and lower obesity rates, and as result their risk of T2D reduced.

In addition to diet, built environment was studied in relation to physical inactivity, an important contributor to insulin resistance. For example, across sectional study enrolled (2026 adults aged 45-84 years); indicated that greater neighborhood physical activity resources were correlated with lower insulin resistance, even after adjusting for age, sex, ethnicity, family history of diabetes, education, and income (Auchincloss, Roux, Brown, Erdmann & Bertoni, 2008). Further, Sidawi and Al-Hariri (2012) underscored that the lack of healthy environments significantly hindered Saudi with T2D from controlling their disease.

In Saudi Arabia, there is a lack of community services such as public gardens, recreation, and sport centers within the neighborhoods. Sidawi et al. (2014) carried out a survey on 76 Saudi patients with T2D; underscored that majority of respondents who had diabetes earlier were complaining from the far distance of fitness centers compared to those who had diabetes later. Additionally, badly designed public gardens and sport centers force Saudi with T2D to experience drowsiness and have difficulties in roaming to and within these centers and parks particularly during the severe hot weather that lasts nearly six months (Sidawi et al., 2014).

## 4. Discussion

The prevalence of T2D will continue to rise in the Saudi population over the next two decades (Alqurashi et al., 2011). The review revealed that Saudi Arabia’s, increase in diabetes rates is related to adapting unhealthy lifestyles including, but not limited to physical inactivity, unhealthy diet, and smoking. Further, the review indicated that lack of education, social support, and healthy environments are barriers to control T2D among the Saudi population. The results of this review underscored that Saudi government has a major task to combat diabetes. Different diabetes risk factors with higher prevalence of these factors put the Ministry of Health (MOH) of Saudi Arabia in a big challenge to curb the burden of diabetes cases. Fortunately, these diabetes risk factors are modifiable (preclude ageing) and can be controlled by adapting healthy lifestyles. These findings can be useful in planning a well public diabetes prevention, which may reduce the length of the journey.

Tourkmani, (2014) recommended that increasing public awareness include delivering public lectures and publication of small Arabic brochures and articles in newspapers about diabetes risk factors are needed to reducing the prevalence of T2D in Saudi Arabia. Even though increasing the level of awareness is important in diabetes prevention, this approach is already in use among the Saudi population. For example, Abdelmarouf et al. (2011) disclosed that Saudi population has enough knowledge of the general knowledge of diabetes regarding risk factors and symptoms. Therefore, alternative approaches include health education, social support, and building healthy environments should be considered to curb T2D in Saudi Arabia.

Health education provided through primary health care centers is a very useful approach for controlling diabetes. Public health providers including primary care physicians, nurses, and dietitians can play an important role in educating their patients by underscoring the importance of dietary intervention, exercise, and weight control. The authors posit that health care providers may not be doing enough to educate the Saudi population.

Abdelmarouf et al. (2011) carried out a cross sectional study on 2007 Saudi citizens and reported that relatives and friends, in addition to media were major sources of information on diabetes (74% and 47% respectively), while healthcare professionals represented the lowest source of information (19%). Therefore, further exploration is needed to determine barriers that health care providers face when educating their patients about T2D in Saudi Arabia. Is it because there is lack of knowledge about how to best disseminate information about diabetes? Is it because of language barriers since many health care providers in Saudi Arabia are foreigners particularly nurses? More studies are needed to elucidate these complex issues.

In addition to health education, social support can play effective role in reducing the burden of T2D in Saudi Arabia. Saudi families should be a part of diabetes treatment and prevention. Educating family members about diabetes risk factors may lead to positive diabetes outcomes. Abdelmarouf et al. (2011) indicated that family and friends are main source of diabetes information in Saudi Arabia. Family members, particularly parents and older brothers have the power to change the family lifestyles in Saudi Arabia. These members are well respected and are also the main supporters of their households in Saudi Arabia. Targeting and educating them about the importance of adapting healthy lifestyles may lead family members to incorporate healthier lifestyle choices.

Beside social support, building future communities that include fitness centers, parks, cycling lanes, and sidewalks are needed to promote healthy lifestyles among the Saudi population. Policies to increase access to grocery stores and limit access to fast food could lead Saudis to have better diets, lower obesity rates, and potentially reduce the risk of T2D. In deed, cooperation between public health providers, clinicians, community leaders, gatekeepers, and stakeholders would be useful to identifying the needs of each community’s access to healthy food and recreation centers. These partnerships may lead to effective diabetes prevention and management to each Saudi community.

### 4.1 Limitations

Some studies included in this review had small sample sizes. Additionally, there were many cross-sectional studies in this review, which limited causal inferences. Also relying solely on self-report may lead to invalid results. Further, most of studies included in this review were conducted in the center, west, and east regions of Saudi Arabia, which limit generalizability.

### 4.2 Future Recommendations

Effective research programs are needed to address the modifiable risk factors and barriers to control T2D targeting Saudi Arabians. Further, understanding community needs is important to combat diabetes; therefore, conducting community based participatory research is needed to address the deficits that prevent residents from controlling their diabetes. Additionally, conducting studies in the north and south regions of Saudi Arabia to identify risk factors for and barrier to control T2D are strongly recommended.

## 5. Conclusion

This review revealed that T2D represents a major public health problem in Saudi Arabia. Epidemiologists, public health researchers, and health policy makers should collaborate to develop comprehensive programs for diabetes prevention and management among the Saudi population. Prospective longitudinal studies with large sample sizes are needed to further address diabetes risk factors for and barriers to control T2D among the Saudi population.

## References

[1] Aida H, Heather G, Maria V, Peter W (2013). Diabetes awareness and behavioural risk factors among university students in Saudi Arabia. Middle East Journal of Family Medicine.

[2] Alanzi T. M, Istepanian R. S. H, Philip N (2014). Usability study of mobile social networking system among Saudi Type 2 diabetes patients (SANAD). Biomedical Engineering (MECBME), 2014 Middle East Conference.

[3] Albakr W, Mohammad A. S, Mohammed A. M, Khamis A. H (2013). Prevalence and Risk Factors of Diabetes Mellitus (I II) in a Sample of Adults Population of Al-Khobar City, Saudi Arabia, within 2010-2011. Life Science Journal.

[4] Al-Daghri N. M, Al-Attas O. S, Alokail M. S, Alkharfy K. M, Yousef M, Sabico S. L, Chrousos G. P (2011). Diabetes mellitus type 2 and other chronic non-communicable diseases in the central region, Saudi Arabia (Riyadh cohort 2): A decade of an epidemic. BMC Medicine.

[5] Alharbi N. S, Almutari R, Jones S, Al-Daghri N, Khunti K, de Lusignan S (2014). Trends in the prevalence of type 2 diabetes mellitus and obesity in the Arabian Gulf States: Systematic review and meta-analysis. Diabetes Research and Clinical Practice.

[6] Al Hayek A. A, Robert A. A, Al Saeed A, Alzaid A. A, Al Sabaan F. S (2014). Factors Associated with Health-Related Quality of Life among Saudi Patients with Type 2 Diabetes Mellitus: A Cross-Sectional Survey. Diabetes Metabolism Journal.

[7] Aljohani N. J (2014). Metabolic syndrome: Risk factors among adults in Kingdom of Saudi Arabia. Journal of Family Community Medicine.

[8] Aljoudi A. S, Taha A. Z (2009). Knowledge of diabetes risk factors and preventive measures among attendees of a primary care center in eastern Saudi Arabia. Annals of Saudi Medicine.

[9] Al-Khudairy L, Stranges S, Al-Dagheri N, Al-Attas O, Alokail M, Al-Kharfy K, Rees K (2014). PP09 Cultural barriers to healthy eating in Saudi adults with and without type 2 diabetes (T2D). Journal of Epidemiology and Community Health.

[10] Al-Khudairy L, Stranges S, Kumar S, Al-Daghri N, Rees K (2013). Dietary factors and type 2 diabetes in the middle east: What is the evidence for an association?--a systematic review. Nutrients.

[11] Allanah L, Ashley K, Farley E (2010). Diabetes and the built environment: Contributions from an emerging interdisciplinary research programme. UWOMJ.

[12] Al-Nozha M. M, Al-Hazzaa H. M, Arafah M. R, Al-Khadra A, Al-Mazrou Y. Y, Al-Maatouq M. A, Al-Shahid M. S (2007). Prevalence of physical activity and inactivity among Saudis aged 30-70 years: A population-based cross-sectional study. Saudi Medical Journal.

[13] AlQuaiz A. M, Tayel S. A (2009). Barriers to a healthy lifestyle among patients attending primary care clinics at a university hospital in Riyadh. Annals of Saudi Medicine.

[14] Alqurashi K. A, Aljabri K. S, Bokhari S. A (2011). Prevalence of diabetes mellitus in a Saudi community. Annals of Saudi Medicine.

[15] Al-Quwaidhi A, Critchley J, O′Flaherty M, Pearce M (2013). Obesity and type 2 diabetes mellitus: A complex association. Saudi Journal of Obesity.

[16] Al-Quwaidhi A. J, Pearce M. S, Critchley J. A, Sobngwi E, O’Flaherty M (2014). Trends and future projections of the prevalence of adult obesity in Saudi Arabia, 1992-2022/[TEXT NOT REPRODUCIBLE IN ASCII]/Tendances et projections de la prevalence de l’obesite chez l’adulte en arabie saoudite, 1992-2022. Eastern Mediterranean Health Journal.

[17] Al-Quwaidhi A. J, Pearce M. S, Sobngwi E, Critchley J. A, O’Flaherty M (2014). Comparison of type 2 diabetes prevalence estimates in Saudi Arabia from a validated Markov model against the International Diabetes Federation and other modelling studies. Diabetes Research and Clinical Practice.

[18] Al-Shahrani A. M, Hassan A, Al-Rubeaan K. A, Al Sharqawi A. H, Ahmad N. A (2012). Effects of diabetes education program on metabolic control among Saudi type 2 diabetic patients. Pakistan Journal of Medical Sciences.

[19] Alsunni A. A, Albaker W. I, Badar A (2014). Determinants of misconceptions about diabetes among Saudi diabetic patients attending diabetes clinic at a tertiary care hospital in Eastern Saudi Arabia. Journal of Family Community Medicine.

[20] Amin T. T, Al Khoudair A. S, Al Harbi M. A, Al Ali A. R (2012). Leisure time physical activity in Saudi Arabia: Prevalence, pattern and determining factors. Asian Pacific Journal of Cancer Prevention: APJCP.

[21] Amin T. T, Al Sultan A. I, Mostafa O. A, Darwish A. A, Al-Naboli M. R (2014). Profile of Non-Communicable Disease Risk Factors among Employees at a Saudi University. Asian Pacific journal of Cancer Prevention: APJCP.

[22] Ansari R. M, Dixon J. B, Browning C. J (2014). Self-Management of Type 2 Diabetes in Middle-Aged Population of Pakistan and Saudi Arabia. Open Journal of Preventive Medicine.

[23] Assaad-Khalil S. H, Al Arouj M, Almaatouq M, Amod A, Assaad S. N, Azar S. T, Alberti K. G. M. M (2013). Barriers to the delivery of diabetes care in the Middle East and South Africa: A survey of 1,082 practicing physicians in five countries. International journal of Clinical Practice.

[24] Auchincloss A. H, Roux A. V. D, Brown D. G, Erdmann C. A, Bertoni A. G (2008). Neighborhood resources for physical activity and healthy foods and their association with insulin resistance. Epidemiology.

[25] Badran M, Laher I (2012). Type II diabetes mellitus in Arabic-speaking countries. International Journal of Endocrinology 2012.

[26] Bakhotmah B. A (2013). Prevalence of obesity among type 2 diabetic patients: Non-smokers housewives are the most affected in Jeddah, Saudi Arabia. Open Journal of Endocrine and Metabolic Diseases.

[27] Bassiony M. M (2009). Smoking in Saudi Arabia. Saudi Medical Journal.

[28] Boutayeb A, Boutayeb W, Lamlili M. E. N, Boutayeb S (2013). Indirect cost of Diabetes in the Arab Region. Int J Diabetol Vasc Dis Res.

[29] Danaei G, Finucane M. M, Lu Y, Singh G. M, Cowan M. J, Paciorek C. J, Ezzati M (2011). Global Burden of Metabolic Risk Factors of Chronic Diseases Collaborating Group (Blood Glucose) National, regional, and global trends in fasting plasma glucose and diabetes prevalence since 1980: Systematic analysis of health examination surveys and epidemiological studies with 370 country-years and 2.7 million participants. Lancet.

[30] Echouffo-Tcheugui J. B, Dagogo-Jack S (2012). Preventing diabetes mellitus in developing countries. Nature Reviews Endocrinology.

[31] El Bcheraoui C, Basulaiman M, Tuffaha M, Daoud F, Robinson M, Jaber S, Mokdad A. H (2014). Status of the diabetes epidemic in the Kingdom of Saudi Arabia, 2013. International Journal of public health.

[32] Eze I. C, Schaffner E, Zemp E, von Eckardstein A, Turk A, Bettschart R, Probst-Hensch N (2014). Environmental tobacco smoke exposure and diabetes in adult never-smokers. Environmental Health.

[33] Ginter E, Simko V (2013). Global prevalence and future of diabetes mellitus. Diabetes.

[34] Guariguata L, Whiting D. R, Hambleton I, Beagley J, Linnenkamp U, Shaw J. E (2014). Global estimates of diabetes prevalence for 2013 and projections for 2035. Diabetes Research and Clinical practice.

[35] Hu FB (2011). Globalization of Diabetes The role of diet, lifestyle, and genes. Diabetes Care.

[36] Jarallah J. S, Al-Rubeaan K. A, Al-Nuaim A. R. A, Al-Ruhaily A. A, Kalantan K. A (1999). Prevalence and determinants of smoking in three regions of Saudi Arabia. Tobacco Control.

[37] Jee S. H, Foong A. W, Hur N. W, Samet J. M (2010). Smoking and risk for diabetes incidence and mortality in Korean men and women. Diabetes Care.

[38] Kadirvelu A, Sadasivan S, Ng S. H (2012). Social support in type II diabetes care: A case of too little, too late. Diabetes, Metabolic Syndrome and Obesity: Targets and Therapy.

[39] Khan A. R, naser al abdul Lateef Z, Fatima S, Al Yousuf S. A. A, Afghan S. Z. K, Al Marghani S (2014). Prevalence of Chronic Complication among Type 2 Diabetics Attending Primary Health Care Centers of Al Ahsa District of Saudi Arabia: A Cross Sectional Survey. Global Journal of Health Science.

[40] Larson N. I, Story M. T, Nelson M. C (2009). Neighborhood environments: Disparities in access to healthy foods in the US. American Journal of Preventive Medicine.

[41] Lee I. H, Park S. Y (2014). Impairment of balance in elderly subjects with type 2 diabetes. Journal of physical Therapy Science.

[42] Lindström J, Peltonen M, Eriksson J. G, Ilanne-Parikka P, Aunola S, Keinänen-Kiukaanniemi S, Finnish Diabetes Prevention Study (DPS) (2013). Improved lifestyle and decreased diabetes risk over 13 years: Long-term follow-up of the randomised Finnish Diabetes Prevention Study (DPS). Diabetologia.

[43] Luo J, Rossouw J, Tong E, Giovino G. A, Lee C. C, Chen C, Margolis K. L (2013). Smoking and diabetes: Does the increased risk ever go away?. American Journal of Epidemiology.

[44] Mahfouz A. A, Shatoor A. S, Al-Ghamdi B. R, Hassanein M. A, Nahar S, Farheen A, Rabie F. M (2013). Tobacco use among health care workers in southwestern Saudi Arabia. BioMed Research International 2013.

[45] Mayberry L. S, Osborn C. Y (2012). Family support, medication adherence, and glycemic control among adults with type 2 diabetes. Diabetes Care.

[46] Memish Z. A, El Bcheraoui C, Tuffaha M, Robinson M, Daoud F, Jaber S, Al Rabeeah A. A, Obesity and associated factors--kingdom of saudi arabia, 2013 (2014). Preventing Chronic Disease.

[47] Midhet F, Al Mohaimeed A, Sharaf F (2010a). Dietary practices, physical activity and health education in qassim Region of Saudi Arabia. International Journal of Health Sciences.

[48] Midhet F, Al-Mohaimeed A, Sharaf F (2010b). Lifestyle related risk factors of type 2 diabetes mellitus in Saudi Arabia. Saudi Medical Journal.

[49] Miller T. A, DiMatteo M. R (2013). Importance of family/social support and impact on adherence to diabetic therapy. Diabetes, Metabolic Syndrome and Obesity: Targets and Therapy.

[50] Mohieldein A. H, Al Zohairy M. A, Hasan M (2011). Awareness of diabetes mellitus among Saudi non- diabetic population in Al-Qassim region, Saudi Arabia. J Diabetes Endocrinol.

[51] Murad M. A, Abdulmageed S. S, Iftikhar R, Sagga B. K (2014). Assessment of the Common Risk Factors Associated with Type 2 Diabetes Mellitus in Jeddah. International Journal of Endocrinology, 2014.

[52] Nam S, Chesla C, Stotts N. A, Kroon L, Janson S. L (2011). Barriers to diabetes management: Patient and provider factors. Diabetes Research and Clinical Practice.

[53] Ng S. W, Zaghloul S, Ali H. I, Harrison G, Popkin B. M (2011). The prevalence and trends of overweight, obesity and nutrition-related non-communicable diseases in the Arabian Gulf States. Obesity Reviews.

[54] Okura T, Heisler M, Langa K. M (2009). Association between cognitive function and social support with glycemic control in adults with diabetes mellitus. Journal of the American Geriatrics Society.

[55] Olokoba A. B, Obateru O. A, Olokoba L. B (2012). Type 2 diabetes mellitus: A review of current trends. Oman Med J.

[56] Pasala S. K, Rao A. A, Sridhar G. R (2010). Built environment and diabetes. International Journal of Diabetes in developing countries.

[57] Qi L, Hu F. B, Hu G (2008). Genes, environment, and interactions in prevention of type 2 diabetes: A focus on physical activity and lifestyle changes. Current Molecular Medicine.

[58] Rawal L. B, Tapp R. J, Williams E. D, Chan C, Yasin S, Oldenburg B (2012). Prevention of type 2 diabetes and its complications in developing countries: A review. International Journal of Behavioral Medicine.

[59] Sacerdote C, Ricceri F, Rolandsson O, Baldi I, Chirlaque M. D, Feskens E, Wareham N (2012). Lower educational level is a predictor of incident type 2 diabetes in European countries: The EPIC-InterAct study. International Journal of Epidemiology.

[60] Saeed A. A (2012). Association of Tobacco Products Use and Diabetes Mellitus-Results of a National Survey Among Adults in Saudi Arabia. Balkan Medical Journal.

[61] Salois M. J (2012). Obesity and diabetes, the built environment, and the ‘local’ food economy in the United States, 2007. Economics Human Biology.

[62] Schiøtz M. L, Bøgelund M, Almdal T, Jensen B. B, Willaing I (2012). Social support and self-management behaviour among patients with Type 2 diabetes. Diabetic Medicine.

[63] Shaw J. E, Sicree R. A, Zimmet P. Z (2010). Global estimates of the prevalence of diabetes for 2010 and 2030. Diabetes Research and Clinical Practice.

[64] Sidawi B, Al-Hariri M. T. A (2012). The impact of built environment on diabetic patients: The case of Eastern Province, Kingdom of Saudi Arabia. Global Journal of Health Science.

[65] Sidawi B, Alhariri M. T, Albaker W. I (2014). Creating a Healthy Built Environment for Diabetic Patients: The Case Study of the Eastern Province of the Kingdom of Saudi Arabia. Global Journal of Health Science.

[66] Sidawi B, Deakin M (2013). Diabetes, built environments and (un) healthy lifestyles: The potential of smart city technologies. Smart and Sustainable Built Environment.

[67] Smith C. A (2012). Living with sugar: Influence of cultural beliefs on Type 2 Diabetes self-management of English-speaking Afro-Caribbean women. Journal of Immigrant and Minority Health.

[68] Smoikng and Diabetes (2014). Centers for Disease Control and Prevention Web site.

[69] Social Determinants of Health (2014). U.S. Department of Health and Human Services.

[70] Temelkova-Kurktschiev T, Stefanov T (2012). Lifestyle and genetics in obesity and type 2 diabetes. Experimental and Clinical Endocrinology diabetes: Official journal, German Society of Endocrinology [and] German Diabetes Association.

[71] Tourkmani A. M (2014). Diabetes mellitus in Saudi Arabia: A major public health concern. Australasian Medical Journal (Online).

[72] Tuomilehto J, Lindström J, Eriksson J. G, Valle T. T, Hämäläinen H, Ilanne-Parikka P, Uusitupa M (2001). Prevention of type 2 diabetes mellitus by changes in lifestyle among subjects with impaired glucose tolerance. New England Journal of Medicine.

[73] Type2 (2015). American Diabetes Association.

[74] Unick J. L, Beavers D, Jakicic J. M, Kitabchi A. E, Knowler W. C, Wadden T. A, Wing R. R (2011). Effectiveness of lifestyle interventions for individuals with severe obesity and type 2 diabetes results from the Look AHEAD trial. Diabetes care.

[75] Weinger K, Beverly E. A, Smaldone A (2014). Diabetes Self-Care and the Older Adult. Western Journal of Nursing Research.

[76] Zhang L, Curhan G. C, Hu F. B, Rimm E. B, Forman J. P (2011). Association between passive and active smoking and incident type 2 diabetes in women. Diabetes Care.

